# Comparative Evaluation of the Cytotoxic Effect of *Dysphania ambrosioides* Extracts Against Human Breast and Cervical Cancer Cells

**DOI:** 10.1002/cbdv.71452

**Published:** 2026-07-09

**Authors:** Andres Camilo Moreno Abadia, Lina Marcela Trujillo Chacón, Júlia Biz Willig, Edison Osorio, Diogo André Pilger, Miriam Anders Apel, Simone Cristina Baggio Gnoatto

**Affiliations:** ^1^ Laboratory of Phytochemistry and Organic Synthesis (LaFiS) Federal University of Rio Grande do Sul Porto Alegre Rio Grande do Sul Brazil; ^2^ Grupo de Investigación en Sustancias Bioactivas Facultad de ciencias farmacéuticas y alimentarias Universidad de Antioquia UdeA Medellín Colombia; ^3^ Laboratory of Biochemical and Cytological Analyses (LABC) School of Pharmacy Federal University of Rio Grande do Sul Porto Alegre Rio Grande do Sul Brazil; ^4^ Pharmacognosy Laboratory Federal University of Rio Grande do Sul Porto Alegre Rio Grande do Sul Brazil

**Keywords:** Apoptosis, Cytotoxicity, Natural products, Phytochemistry

## Abstract

*Dysphania ambrosioides*, a prominent medicinal plant, exhibits diverse biological properties with emerging potential in cancer research. Despite its therapeutic promise, the relationship between phytochemical composition and biological activities remains insufficiently explored, particularly regarding geographical variation. This study investigates the correlation between the phytochemical profiles of *D. ambrosioides* extracts from Colombia (DA‐Col) and Brazil (DA‐Bra) and their cytotoxic activity against HeLa, SiHa, and MCF‐7 cancer cell lines. Qualitative chemical profiling revealed distinct presence‐and‐absence patterns of specific secondary metabolites between the two geographical sources. These geographically driven variations in the phytochemical profile directly influenced the observed biological activity, with the extracts exhibiting differential cytotoxicity profiles. Specifically, Colombian extract DA‐Col‐1 showed significant activity against HeLa cells (IC_50_ = 3.09  ± 0.32 µg/mL) and DA‐Col‐2 demonstrated potent effects on MCF‐7 cells (IC_50_ = 2.08  ± 0.58 µg/mL). Brazilian extract DA‐Bra‐2 selectively inhibited MCF‐7 proliferation (IC_50_ = 3.09  ± 0.12 µg/mL). Apoptosis assays indicated that these extracts might induce early apoptotic events, suggesting a potential mechanism of action that warrants further investigation. This study establishes a direct link between the chemical composition of geographically distinct *D. ambrosioides* samples and their selective anticancer potential, providing a foundation for natural product‐based drug development targeting specific cancer types.

## Graphical abstract

This study evaluates the geographic influence on the chemical and biological profiles of *Dysphania ambrosioides* collected from Brazilian and Colombian sources. Following extraction, metabolic fingerprinting via HPLC‐MS reveals distinct phytochemical variations between the geographical origins. Furthermore, *in vitro* evaluations demonstrated that these extracts possess significant cytotoxic activity against HeLa, SiHa, and MCF‐7 cancer cell lines, establishing a link between provenance and therapeutic potential.

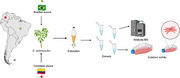

**Abbreviations**
DA‐Bra: (*D. ambrosioides—*Brazil)
DA‐Bra‐1: (medium polarity extract from DA‐Bra)
DA‐Bra‐2: (polar extract from DA‐Bra)
DA‐Col: (*D. ambrosioides*—Colombia)
DA‐Col‐1: (medium polarity extract from DA‐Col)
DA‐Col‐2: (polar extract from DA‐Col)

## 1 Introduction

Historically, plants represent fundamental therapeutic agents, embodying an essential component of health systems and a profound cultural legacy [1]. This is particularly evident in how herbal products have been crucial in traditional medicine, significantly contributing to contemporary phytotherapeutic knowledge [2]. In this context, ethnopharmacology has been instrumental in documenting indigenous medical wisdom and elucidating the associated biodiversity [3]. Through this documentation, Natural products, characterized by their diverse chemical structures, have demonstrated substantial effects as biological function modulators, attracting significant scientific interest [4]. Crucially, traditional usage practices and plant preparation techniques provide valuable guidance for laboratory extraction methodologies and experimental designs [5]. Despite this potential, the vast majority of medicinal plants remains extensively unexplored, with approximately half a million species worldwide, most of which have not been comprehensively investigated for their therapeutic properties [6].

Among these plants, *Dysphania ambrosioides* stands out for its global distribution and is recognized by the World Health Organization as one of the most widely used medicinal plants, with well‐documented antimicrobial, anti‐inflammatory, and antiparasitic properties [7]. Native to South and Central America, the plant is also prevalent in Africa, Europe, Australia, and various Asian countries [7, 8]. Commonly known as “wormseed”, “Mexican tea”, or “epazote”, *D. ambrosioides* belongs to the Amaranthaceae family [9].

Ethnopharmacological investigations across different regions reveal remarkably consistent therapeutic applications of *D. ambrosioides*, particularly in addressing intestinal infections and inflammatory conditions [10]. This regional consistency is complemented by a wide spectrum of specific local uses; for instance, in Colombia, the plant is employed to treat conditions ranging from asthma and digestive disorders to malaria and spasms, while in Brazil, it is utilized for addressing muscle pain, inflammatory conditions, and as an antimicrobial agent [11, 12]. Beyond these traditional applications, Emerging investigations have highlighted the the plant's potential in cancer research, with studies demonstrating activity against various cancer cell lines such as SMMC‐7721 and P388 leukemia cells [13, 14].

To date, several studies focused on this plant have demonstrated the antiproliferative potential of its essential oil, which has been predominantly employed as the primary matrix in biological investigations. Specifically, this essential oil exhibits a concentration‐ and time‐dependent cytotoxic effects in MCF‐7 cells (IC_50_ = 9.45 µg/mL observed after 24 h) and in RAJI cells (IC_50_ = 1 µg/mL) [15, 16]. Moreover, it has been shown to induce cytotoxicity in normal human liver cells (L02), causing cell cycle arrest at the S phase and triggering apoptosis through the intrinsic mitochondrial pathway [17].

Collectively, these findings highlight the promising biological potential of *D. ambrosioides* as a source of novel anticancer agents. However, current research has focused predominantly on the use of the essential oil, thereby limiting exploration to this single matrix and overlooking other biological fractions, such as aqueous and organic extracts. As a result, studies involving these alternative matrices remain scarce. Compounding this issue, the plant's phytochemical composition is significantly influenced by geographical and environmental factors, including altitude, precipitation, and temperature, which can modify the concentration of active compounds and consequently alter biological activity [18]. This variability underscores the necessity of comparative analyses across different populations. Therefore, to address these gaps, this study aims to investigate the correlation between the phytochemical composition of *D. ambrosioides* extracts from Colombia (DA‐Col) and Brazil (DA‐Bra) and their biological activity against HeLa, SiHa, and MCF‐7 cancer cell lines.

## 2 Experimental Section

### 2.1 Plant Material

The leaves of *D. ambrosioides* (DA‐Bra) were collected in Siderópolis, SC, Brazil, coordinates 28°35′59.0″S 49°25″54.2″W. They were subsequently transferred to the Faculty of Pharmacy at the Federal University of Rio Grande do Sul. The leaves of *D. ambrosioides* (DA‐Col) were collected in the rural area of Alto Baudó municipality, Chocó, Colombia in the district of Chachajo, coordinates 5° 42′ 0″ N 77° 0′ 0″ W. The samples were taken to the Grupo de Investigación en Sustancias Bioactivas (GISB) of the University of Antioquia. Both samples were collected on January 2024. Taxonomic identification of both plant materials was performed by Vanilde Citadini Zanette and voucher specimens were deposited under numbers 205746 (for DA‐Bra) and 224043 (for DA‐Col) in the herbaria of the Federal University of Rio Grande do Sul and the University of Antioquia, respectively.

### 2.2 Extraction

The plant material was dehydrated at 40°C for 48 h and pulverized (DA‐Col 30.45 ± 0.15 g, DA‐Bra 30.75 ± 0.35 g). The pulverized plant material separately subjected to a mixture of 200 mL of methanol/dichloromethane (Sigma–Aldrich, Vienna, Austria) in equal parts as extraction solvent, to be subsequently extracted in an ultrasonic bath in an Elmasonic P60H ultrasound (Elma Schmidbauer GmbH, Elmasonic P60H, Singen, Germany) for 15 min, operating at 37 kHz, 180 Watts and 40°C. Then, the solvents were separated from the plant residue, repeating the previous procedure two more times, completing a total time of 45 min. Subsequently, the solvents from each of the extractions were combined, reducing their volume to 100 mL. Subsequently, a final extraction was performed on the resulting plant residues in 100 mL of methanol, finally joining the solvent from this extraction with the previously reduced solvents. Then, this mixture was taken to dryness by reduced pressure using a rotary evaporator (Buchi Labortechnik AG, Switzerland), obtaining the solid products corresponding to the extracts of moderately polar compounds. This process was repeated for both DA‐Col and DA‐Bra independently, obtaining as products DA‐Col‐1 (Medium polarity extract from DA‐Col) and DA‐Bra‐1 (Medium polarity extract extract from DA‐Bra).

To obtain the polar compounds, the plant residues resulting from the previous procedure were taken and subjected to extraction with 500 mL of hot water, allowing it to boil for 15 min. After the mixture had cooled, it was filtered to be subsequently treated with previously activated Amberlite XAD‐7 (Sigma–Aldrich, Vienna, Austria), under stirring for 1 h. Consecutively, after the mixture was decanted, two successive washes were performed to eliminate sugars and salts. Finally, the compounds retained in the resin were reabsorbed by adding 150 mL of methanol twice, being taken to dryness until reaching the solid product corresponding to the polar extracts. This procedure was repeated with residues from the first extraction of DA‐Col and DA‐Bra, obtaining DA‐Col‐2 (Polar extract from DA‐Col) and DA‐Bra‐2 (Polar extract from DA‐Bra). The yields for DA‐Col‐1, DA‐Bra‐1, DA‐Col‐2, and DA‐Bra‐2 were 0.2%, 0.082%, 0.080%, and 0.12%, respectively.

### 2.3 Phytochemical Profiling of Plant Extracts by UHPLC‐QTOF‐MS

Extracts analysis was carried out using a UHPLC‐QTOF‐MS system, UHPLC (Shimadzu‐Nexera x2,) equipped with a Shim‐pack XR‐ODS III column (2.0 × 50 mm, 1.6 µm) from Shimadzu thermostated at 35°C coupled to the QTOF‐MS mass analyzer (Impact II, Bruker Daltonics). The QTOF‐MS system was equipped with an electrospray ionization (ESI) source, operating in positive and negative ionization mode. The adopted elution gradient mode, the mobile phase consisted of A: methanol (0.1% formic acid) and B: aqueous phase (0.1% formic acid). The elution gradient started at 5% of A maintained for 2 min, increased to 95% in the next 10 min, and kept for 3 min. Then 95% A linearly decreased to 5% in 2 min, kept for 5 min. The flow rate was 0.3 mL min^−1^ and the injection volume was 5 µL. The operation parameters of ESI were the following: negative mode, capillary voltage, 2500 V; end plate offset, 500 V; nebulizer pressure, 3 bar (N_2_); drying gas, 9 L min^−1^ (N_2_); and drying temperature, 200°C. In positive mode, capillary voltage, 4000 V; end plate offset, 500 V; nebulizer pressure, 3 bar (N2); drying gas, 9 L min^−1^ (N_2_); and drying temperature, 190°C. The QTOF‐MS system was operating in broadband collision‐induced dissociation (bbCID) acquisition mode and recorded spectra over the range *m*/*z* 55−1600 with a scan rate of 2 Hz. This mode provides MS and MS/MS spectra at the same time, working at two different collision energies; at low collision energy (10 eV), MS spectra were acquired. At high collision energy (70 eV), no isolation took place at the quadrupole, and the ions from the preselected mass range are fragmented at the collision cell. A QTOF‐MS external calibration was performed before each injection with a sodium formate solution. Data treatment was processed with Data Analysis 4.2 Software. Besides the accurate mass measurement (error < 5 ppm).

### 2.4 Determination of Cell Viability

#### 2.4.1 Cell Culture

The cells used in this study: HeLa (human cervical adenocarcinoma; RRID:CVCL_0030), SiHa (human cervical adenocarcinoma—HPV‐16; RRID:CVCL_0032), MCF‐7 (human breast adenocarcinoma; RRID:CVCL_0031) were obtained from American Type Culture Collection (ATCC—Rockville, MD, USA) and Vero (Green African monkey kidney cell) were obtained from Rio de Janeiro Cell Bank (RJ, Brazil). The cell lines were maintained in a culture medium supplemented with 10%—(*v*/*v*) fetal bovine serum (Gibco, Grand Island, NY, USA), 100 U/mL penicillin (Sigma–Aldrich, St. Louis, MO, USA) and 100 µg/mL streptomycin (Sigma–Aldrich, St. Louis, MO, USA), 2 mM L‐glutamine (Sigma–Aldrich, St. Louis, MO, USA), at 37°C and in a humidified atmosphere (5% CO_2_). The cell lines of SiHa, HeLa and MCF‐7 were cultivated in DMEM (Dulbecco's Modified Eagle Medium—Sigma) low‐glucose and Vero cells in DMEM high‐glucose (Sigma, St Louis, USA).

#### 2.4.2 Viability Assessment Following Exposure to Plant Extracts

The cytotoxic effect of the compounds on cell lines was performed using 3‐(4,5‐dimethylthiazol‐2‐yl)‐2,5‐diphenyltetrazolium bromide (MTT, Sigma–Aldrich, St. Louis, MO, USA) assay. Each cell line was seeded in 96‐well plates (5 × 10^3^ cancer cells per well) and incubated at 37°C and 5% CO_2_. After achieving semi‐confluence, the cells were treated with compounds at 10 µg/mL in a preliminary screening and incubated for 48 h. The compounds that attained inhibition > 50% were selected to evaluate their concentration‐response using 6 concentrations varying from 0.5 to 50 µg/mL. After 48 h, the medium containing the treatment was removed and the cells were incubated with MTT solution (0.5 mg/mL) for 3 h at 37°C, in the absence of light. Formazan crystals formed were dissolved in DMSO and quantified by absorbance in 570 and 630 nm as measured in a plate spectrophotometer (Spectramax M2e, SoftMax Pro Software Interface 5.4.1, USA). Results are expressed as the percentage of the control. Dose‐response curves were constructed and IC_50_ values were determined by Graphpad Prism software (version 8.0). Vero cells were treated to assess the selectivity index (SI) to evaluate how selective the compound is for killing/damaging cancer cells instead of normal cells. The degree of selectivity of the compound was expressed for each tumor cell line, according to the equation, where selectivity index SI was calculated as the ratio of 50% cytotoxic concentration (CC_50_) to the 50% inhibitory concentration (IC_50_).

#### 2.4.3 Quantitative Assessment of Apoptosis by Annexin v/Propidium Iodide Dual Staining

Phosphatidylserine (PS) externalization was determined by the annexin fluorescence signal of an annexin V–fluorescein isothiocyanate conjugate (Quatro G Pesquisa & Desenvolvimento, Porto Alegre, Brazil), according to the manufacturer's protocol. Briefly, cells were treated at the corresponding concentrations (IC_50_ concentrations for each cell line) for 48 h, then harvested, centrifuged for 5 min at 1500 rpm and the supernatants were discarded. Pellets obtained were re‐suspended with 150 µL of annexin binding buffer. Cells were stained with 2 µL annexin V and 15 µL propidium iodide for 15 min at room temperature in the dark. Additionally, a control without any treatment was tested, and cells treated with cisplatin at a concentration of 100 µM served as the positive control. The apoptotic index was immediately determined on a FACSVerse flow cytometer (BD Biosciences, San Jose, CA, USA).

#### 2.4.4 Statistical Analysis

Experiments were performed three times (*n* = 3) with samples in triplicate, and the results were analyzed by analysis of variance (ANOVA), followed by the Tukey test. The software used was GraphPad Prism version 8.0. (GraphPad Software, San Diego, CA, USA) data analysis system. In addition, multivariate principal component (PCA) statistical analyses were performed using the Python software package (Wilmington, Delaware, USA). Significant values were considered for *p* < 0.05.

## 3 Results

### 3.1 Ultra‐High‐Performance Liquid Chromatography‐Mass Spectrometry (UHPLC‐QToF‐MS) Analysis of Leaves Extracts of *D. ambrosioides*

The results of the analysis of the two populations of *D. ambrosioides*, designated Colombia (DA‐Col) and Brazil (DA‐Bra) showed that the polar extracts (DA‐Col‐2, DA‐Bra‐2) and moderately polar extracts (DA‐Col‐1, DA‐Bra‐1) analyzed by UHPLC‐QToF‐MS contain various derivatives of quercetin, kaempferol and apigenin, along with other phenolic compounds. The identified peaks are shown in Table 1, in which we report the retention time, ESI mass spectrometry in negative and positive ion mode for all the compounds detected. These assignments are confirmed by the analytical data shown in Figures S1–S4. Seventeen compounds were identified, which were analyzed according to the molecular formula and mass‐to‐charge ratio (*m*/*z*). Glycosylated flavonoids were primarily identified, with a predominance of the flavonol class, especially those derived from kaempferol (eight compounds) and quercetin (four compounds). Apigenin derivatives (two compounds), hydroxybenzoic acids derivatives (one compound) and feruloyl hexoside were also identified. Moreover, during MS analyses, flavonoid hexose molecules undergo a unique fragmentation pattern which is characterized mainly by their tendency to readily lose the sugar moieties. Therefore, in O‐glycosyl flavonoids the cleavage takes place at the glycosidic O linkage, leading to the glycans’ elimination, allowing identification of the type of sugar chain attached (monosaccharide, disaccharide, etc.) (compound 1).

**TABLE 1:** Phytochemical profile of flavonoid and polyphenolic compounds identified by mass spectrometry analysis in Colombian extracts (DA‐Col‐1, DA‐Col‐2), and Brazilian extracts (DA‐Bra‐1, DA‐Bra‐2).

N°	Formula	*t*_R_ (min)	*m*/*z* [M+H] or [M‐H]	Molecular weight (g/mol)	MS/MS fragments	Ionization mode	Compound	Aglycone	Extracts			
									DA‐Col‐1	DA‐Col‐2	DA‐Bra‐1	DA‐Bra‐2
1	C_13_H_16_O_9_	4.3	315.0722	316.26	315.0711	−	Dihydroxybenzoic acid hexoside	−	**+**	**+**	+	+
2	C_33_H_40_O_20_	5.1	755.2040	756.66	609, 593, 409.1711	−	Quercetin 3‐O‐[α‐L‐rhamnopyranosyl (1→2)‐β‐D‐ ‐D‐glucopyranoside]‐7‐O‐α‐L‐rhamnopyranoside	Quercetin	−	−	−	**+**
3	C_16_H_20_O_9_	5.3	355.1035	356.32	193.0516	−	Feruloyl Hexoside	−	**+**	−	**+**	−
4	C_27_H_30_O_15_	5.8	593.1512	594.52	431.1930, 385.1889	−	Kaempferol Hexoside‐rhamnoside	Kaempferol	−	**+**	−	−
5	C_26_H_28_O_15_	5.9	581.1501	580.49	241.2024, 449.1066	+	Kaempferol Pentosyl‐hexoside	Kaempferol	**+**	**+**	−	−
6	C_33_H_40_O_20_	6.1	755.2040	756.66	505.1174	−	Quercetin 3‐rutinoside 7‐rhamnoside	Quercetin	**+**	**+**	−	−
7	C_27_H_30_O_16_	6.1	611.1607	610.52	303.0491, 465.1024	+	Kaempferol 3‐gentiobioside	Kaempferol	**+**	**+**	−	−
8	C_21_H_20_O_10_	6.2	433.1129	432.38	342.1319	+	Kaempferol‐O‐deoxyhexoside	Kaempferol	**+**	+	−	−
9	C_27_H_30_O_15_	6.3	593.1512	594.52	579.1350, 393.1766	−	Kaempferol‐3‐O‐rutinoside	Kaempferol	**+**	**+**	−	**+**
10	C_21_H_20_O_11_	6.3	449.1078	448.38	371.2048, 123.1160	+	Quercetin‐7‐O‐rhamnoside	Quercetin	**+**	**+**	−	−
11	C_27_H_30_O_16_	6.4	611.1607	610.52	303, 465, 247, 153	+	Quercetin‐3‐O‐β‐glucopyranosyl‐7‐O‐α‐rhamnopyranoside	Quercetin	**+**	**+**	−	−
12	C_26_H_28_O_15_	6.5	581.1501	580.49	579.1350, 393.1766	+	Kaempferol 3‐lathyroside	Kaempferol	−	−	−	**+**
13	C_27_H_30_O_15_	6.6	593.1512	594.52	415.1976, 301.0715	−	Apigenin‐diglucoside	Apigenin	**+**	**+**	−	−
14	C_21_H_20_O_10_	6.6	433.1129	432.38	433.1113, 284.1263	+	Apigenin‐7‐O‐glucoside	Apigenin	**+**	+	−	−
15	C_21_H_20_O_11_	6.7	449.1078	448.38	371.2048, 123.1160	+	Kaempferol‐3‐O‐β‐glucoside	Kaempferol	**+**	−	−	−
16	C_21_H_20_O_10_	7.0	433.1129	432.38	175.0590, 109.0640	+	Kaempferol‐7‐rhamnoside	Kaempferol	**+**	+	−	−
17	C_16_H_12_O_7_	7.9	315.0510	316.26	227.1281, 183.1387	−	Isorhamnetin	Quercetin	**+**	−	**+**	−

**Abbreviations**: ‐, absent; +, present; RT, retention time.

*Identification of quercetin derivatives*: Compounds 2, 6, 10, and 11 were recognized as quercetin derivatives. The compound 2 (*t*_R_ = 5.2 min) was identified as quercetin 3‐*O*‐[α‐L‐rhamnopyranosyl (1→2)‐β‐D‐ glucopyranoside]‐7‐*O*‐α‐L‐rhamnopyranoside with the molecular formula C_33_H_40_O_20_ with a precursor ion at m/z 755.2040 [M − H] − in the DA‐Bra‐2 extract. The fragment ions obtained were due to the elimination of a fragment ions obtained at *m*/*z* 593 [M − H − 162] − was detected, corresponding to the loss of glucose, and *m*/*z* 609 [M − H − 146] − a rhamnose in the structure. The compound 6 (*t*_R_ = 6.1 min) was suggested to be quercetin 3‐rutinoside 7‐rhamnoside with the molecular formula C_33_H_40_O_20_ and a precursor ion at *m*/*z* 755.2040 [M − H] −, the compound 10 (*t*_R_ = 6.3 min) as quercetin‐7‐O‐rhamnoside C_21_H_20_O_11_ with a precursor ion at m/z 449.1078 [M − H] +, and the compound 11 (t_R_ = 6.4 min) as quercetin‐3‐O‐β‐glucopyranosyl‐7‐O‐α‐rhamnopyranoside C_27_H_30_O_16_ with a precursor ion at m/z 611.1607 [M − H] +. The fragment ions obtained were due to the elimination of a fragment ions obtained at m/z 609, 303 and 465 [M − H – 146,] + corresponding to a rhamnose C_6_H_10_O_4_ in the structure of the three compounds, respectively. The three compounds were detected in the DA‐Col‐1 and DA‐Col‐2 extracts. The qualitative chromatographic analysis also revealed the presence of a few minor, unidentified peaks. These trace constituents were uniquely distributed and varied depending on the geographical origin (DA‐Col or DA‐Bra) of the samples.

**FIGURE 1:**
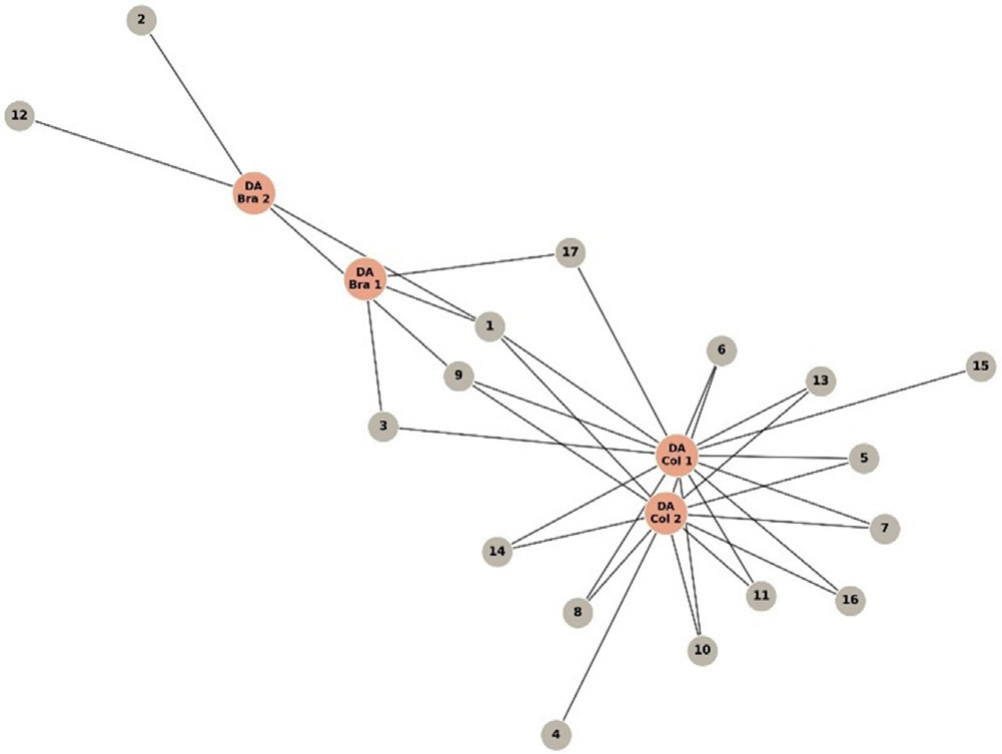
Correspondence network analysis.

The correspondence network analysis (Figure 1) revealed a clear differentiation between the Colombian (DA‐Col) and Brazilian (DA‐Bra) extracts, highlighting significant variations in their phenolic profiles. The Colombian extracts (DA‐Col‐1 and DA‐Col‐2) exhibited a higher number of associations with the identified phenolic compounds, suggesting a more diverse and heterogeneous chemical composition, possibly related to environmental factors or differences in the biosynthetic pathways of the source material. In contrast, the Brazilian extracts (DA‐Bra‐1 and DA‐Bra‐2) formed a more compact cluster with fewer connections, indicating a more homogeneous phenolic composition. The few shared nodes between both groups correspond to common phenolic compounds, which may represent central or structurally conserved metabolites. Overall, the distribution pattern supports the influence of geographical origin on the chemical diversity and phenolic composition of the analyzed extracts.

The phytochemical composition identified in this study shows concordance with previous research [19], through LC‐MS/MS‐QToF analysis of the methanolic extract of *D. ambrosioides*, demonstrated a similar predominance of phenolic compounds, specifically flavonoids derived from kaempferol and quercetin. Similarly, another research when analyzing the aqueous extract of the same species, reported a comparable chemical distribution, characterized by the presence of quercetin and kaempferol‐derived flavonoids, as well as other acids conjugated to flavonoids [20]. These findings corroborate the consistency in the phytochemical profile regardless of the extraction method employed.

The differences in chemical composition are likely influenced by the distinct environmental conditions of the plant origins [18], with Colombia's tropical rainforest and Brazil's Atlantic Forest providing different stress factors that impact metabolite biosynthesis. These findings highlight how geographic and environmental factors influence the phytochemical diversity and bioactivity of plant‐derived extracts, underlining the importance of standardizing extraction conditions to ensure consistent therapeutic effects.

### 3.2 Assessment of Antiproliferative and Cytotoxic Effects

The four extracts of *D. ambrosioides* were evaluated in three cell lines: MCF‐7, HeLa, SiHa, and Vero cells as control. These lines are commonly employed as models of female‐related neoplasms, with HeLa and SiHa being additionally associated with HPV infection. In the initial phase, a general screening was conducted at concentrations of 1, 10, 25, and 50 µg/mL. The best response, corresponding to the optimal concentration, was identified at 10 µg/mL (Table 2). This preliminary screening at a standardized concentration of 10 µg/mL for each extract provides essential baseline information on their cytotoxic potential. The extracts DA‐Col‐1 and DA‐Col‐2 exhibited high activity against the HeLa cell line, with cell viability percentages below 30%. For the MCF‐7 cell line, the extracts DA‐Col‐1, DA‐Col‐2, and DA‐Bra‐2 were active, with cell viability percentages below 50%. Accordingly, the extracts considered the most promising and selected for subsequent studies were DA‐Col‐1, DA‐Col‐2, and DA‐Bra‐2; in contrast DA‐Bra‐1 did not display a favorable profile. Although the extracts demonstrated activity against SiHa cells, the results were not considered significant, as cell viability remained above 50%, which does not meet the threshold for promising cytotoxic activity against this cell line.

**TABLE 2:** Percentage of cell viability of different cell lines exposed to *D. ambrosioides* extracts at a concentration of 10 mg/mL.

Extracts	Cell viability (%)			
	Vero	MCF‐7	SiHa	HeLa
DA‐Col‐1	48.72 ± 0.01	**49.37 ± 0.03**	55.35 ± 0.02	**22.95 ± 0.01**
DA‐Col‐2	93.34 ± 0.01	**40.09 ± 0.02**	80.49 ± 0.03	**29.71 ± 0.04**
DA‐Bra‐1	38.38 ± 0.03	56.02 ± 0.01	97.81 ± 0.03	58.42 ± 0.03
DA‐Bra‐2	98.35 ± 0.01	**42.50 ± 0.01**	75.77 ± 0.06	79.85 ± 0.04

*Note*: The results correspond to the mean ± SEM (*n* = 3). Results under 50% of viability have been highlighted.

The three selected extracts were evaluated at six different concentrations (0.5, 1, 5, 10, 25, and 50 µg/mL) on the above‐mentioned cell lines to determine the corresponding half‐maximal inhibitory concentration (IC_50_) and selectivity index from Vero cells (SI) values (Table 3). Determining SI for natural products is important in terms of their clinical use perspective, with a view to greater target selectivity and fewer adverse effects [21]. For compounds with anticancer activity, an SI value ≥ 10 is recommended for studies in increasingly complex models. Based on this criterion, extracts meeting this requirement were selected to evaluate the mode of cell death, specifically examining apoptotic and necrotic pathways. In the MCF‐7 cell line, extracts DA‐Col‐2 and DA‐Bra‐2 exhibited IC_50_ values of 2.08 ± 0.58 and 3.09 ± 0.12 µg/mL, respectively, with selectivity indices of 20.65 and 14.60. These results demonstrate high cytotoxicity and selectivity for both extracts. In contrast, extract DA‐Col‐1 presented an IC_50_ of 5.53 ± 0.72 µg/mL; however, its selectivity index did not reach the threshold value of 10. In the HeLa cell line, extracts DA‐Col‐1 and DA‐Col‐2 recorded IC_50_ values of 3.09 ± 0.32 and 4.40 ± 0.85 µg/mL, with selectivity indices of 16.09 and 9.76, respectively. These values indicate high cytotoxicity and selectivity, with DA‐Col‐1 being the extract that demonstrated the highest selectivity in the HeLa cell line.

**TABLE 3:** IC_50_ values of *D. ambrosioides* extract against MCF‐7 and HeLa cancer cell lines and Vero cell.

Extract	IC_50_ (µg/mL)				
	MCF‐7	SI	HeLa	SI	Vero
DA‐Col‐1	5.53 ± 0.72	8.99	3.09 ± 0.32	**16.09**	49.72 ± 1.58
DA‐Col‐2	2.08 ± 0.58	**20.65**	4.40 ± 0.85	9.76	42.96 ± 0.78
DA‐Bra‐2	3.09 ± 0.12	**14.60**	—	—	45.35 ± 1.25

*Note*: Concentrations ranged from 0.5 to 50 µg/mL in increments of 0.5 µg/mL. Values are the mean ± SD (*n* = 3). SI Results above 10 have been highlighted.

### 3.3 Quantitative Assessment of Apoptosis by Annexin v/Propidium Iodide Dual Staining

**FIGURE 2:**
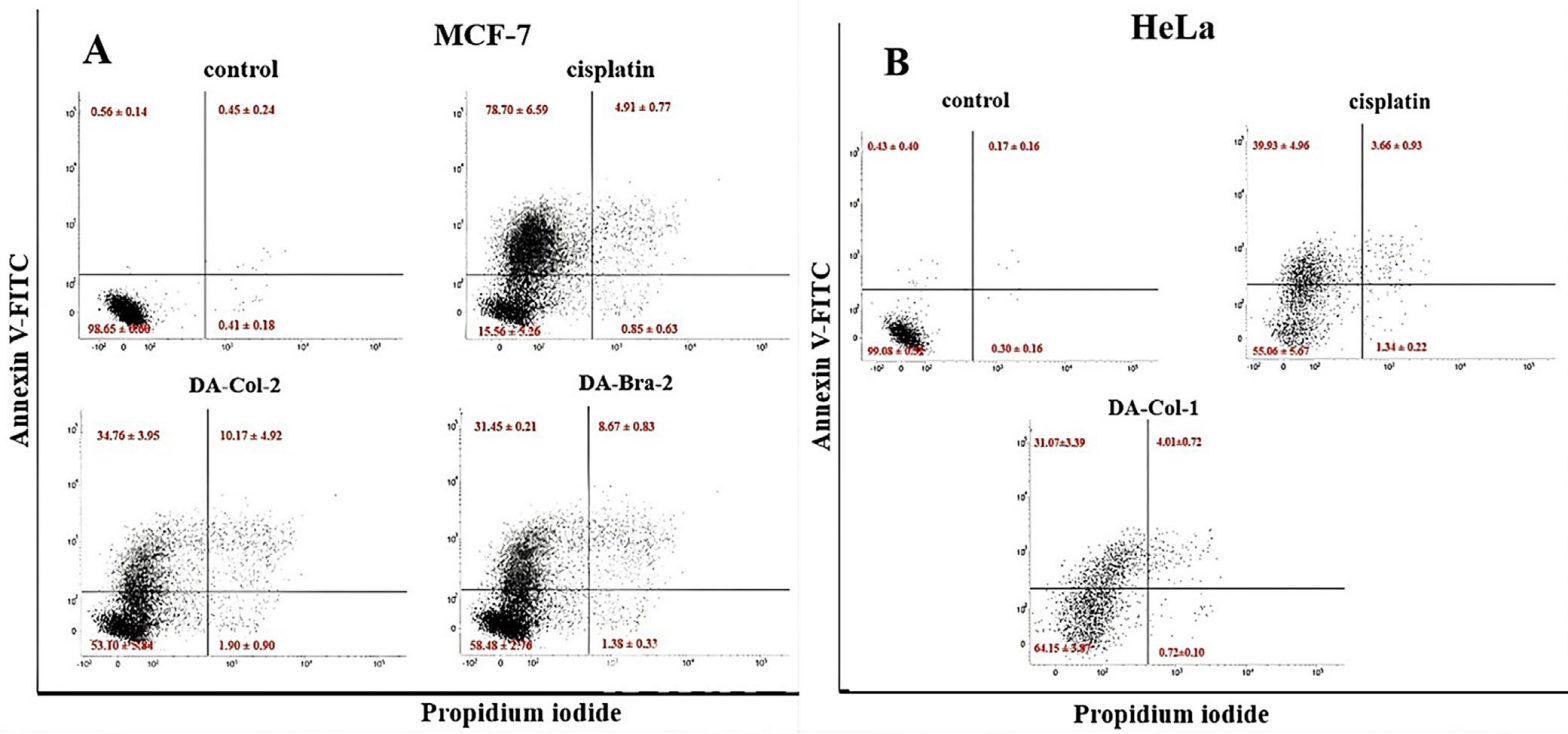
Graphical behavior of cell viability for each extract in the different cell lines using Annexin V‐FITC assay. (A) MCF‐7 after exposure to DA‐Col‐2 and DA‐Bra‐2. (B) HeLa after exposure to DA‐Col‐1 using Annexin V‐FITC assay. Data are reported as mean ± standard deviation (SD) of three experiments (*p < 0.05; relative to untreated control cells).

The extracts demonstrated significant activity in inducing early apoptotic cell death. Cell viability decrease and apoptosis induction following treatment were analyzed using the Annexin V‐FITC assay, and the results are presented in Figure 2. The extracts DA‐Col‐2 and DA‐Bra‐2 evaluated in the MCF‐7 cell line showed early apoptosis rates of 34.76 ± 3.95% and 31.45 ± 0.21%, respectively. The corresponding late apoptosis rates were 10.17 ± 4.92% and 8.67 ± 0.83%, respectively, while necrosis rates were 1.90 ± 0.90% and 1.38 ± 0.33%, respectively. The DA‐Col‐1 extract evaluated in HeLa cells demonstrated an early apoptosis rate of 31.07 ± 3.39%, a late apoptosis rate of 4.01 ± 0.72%, and a low necrosis rate of 0.30 ± 0.16%.

## 4 Discussion

The geographically and polarity distinct four extracts (DA‐Col‐1, DA‐Col‐2, DA‐Bra‐1, and DA‐Bra‐2) were evaluated against breast cancer (MCF‐7), as well as cervical (HeLa and SiHa) cell lines through a cell viability screening, revealing promising aspects regarding their cytotoxic potency. For the MCF‐7 cell line, the extracts DA‐Col‐1, DA‐Col‐2, and DA‐Bra‐2 were active, with cell viability percentages below 50%.

Extract DA‐Bra‐2 exhibited notable selectivity toward the MCF‐7 cell line, reducing its viability below 40% while maintaining the viability of Vero cells close to 100% with a SI of **14.6** and IC_50_ = 3.09 µg/mL. This selectivity is a significant finding, considering that one of the main challenges in developing anticancer agents is precisely the ability to selectively affect tumor cells while preserving normal cell viability. Furthermore, the extracts DA‐Col‐1 and DA‐Col‐2 exhibited high activity against the HeLa and MCF‐7 cells line, with cell viability percentages below 50%. The IC_50_ determination revealed that extract DA‐Col‐2 demonstrated marked selectivity against MCF‐7 cell line at a relatively low concentration (2.08 µg/mL), while simultaneously maintaining a favorable safety profile in Vero cells (SI = operating through **20.65**). Concurrently, DA‐Col‐1 showed significant activity against the HeLa cell line exhibiting an IC_50_ = 3.09 µg/mL and SI = **16.09**.

The observed cytotoxic activity can be attributed to the synergistic action of the characterized phytochemicals, each operating through specific and well‐documented mechanisms of action. *The flavonoids* present in the studied plant extracts exhibit anticancer activity, a widely recognized feature of this group of secondary metabolites and their derivatives. Scientific literature shows that these compounds exert multiple antineoplastic effects through the modulation of enzymes involved in scavenging reactive oxygen species (ROS), cell cycle regulation, the induction of apoptosis and autophagy, as well as inhibition of cell proliferation and invasiveness. Futhermore, flavonoids demonstrate a notable functional duality in ROS homeostasis, acting as antioxidants under normal physiological conditions and as pro‐oxidants in cancer cells, where they activate apoptotic pathways and reduce pro‐inflammatory signaling [22]. This mechanistic versatility supports their potential as chemopreventive and therapeutic agents [23].

*Quercetin* as an aglycone and its glycosylated forms such as quercetin 3‐O‐glucoside, quercetin 7‐O‐glucoside, isorhamnetin, among others [24], have demonstrated significant activity against MCF‐7 cells at concentrations of 25 µmol/mL through the increase of ROS and malondialdehyde (MDA), as well as the modulation of pro‐ and anti‐apoptotic proteins [25]. This activity is confirmed in subsequent studies, where quercetin aglycone isolated from *Acalypha indica L*. showed an IC_50_ of 10 µg/mL against MCF‐7 [26], and effectiveness against HeLa significantly inhibiting cell growth and inducing apoptosis *in vitro* in a time and dose‐dependent manner with at 25 µmol/L [27]. Also, a current study showed cytotoxic activity against HeLa cells at concentrations between 2.5 and 5 µM [28].

*Kaempferol* and its glycosylated derivatives such as Kaempferol‐3‐O‐rhamnoside, contribute significantly to the cytotoxic activity of the studied extracts through distinct but complementary molecular mechanisms [29]. For example, it has been determined the remarkable activity of Kaempferol‐7‐O‐β‐D‐glucoside toward HeLa cell line with an IC_50_ of 42.1 ± 3.2 µg/mL [30]. On the other hand, was documented Kaempferol ability to induce apoptosis and cellular senescence in HeLa through the regulation of PI3K/Akt and hTERT pathways, with IC_50_ values of 45.63, 22.87, and 10.48 mM at 24, 48, and 72 h, respectively [31]. These findings are complemented by the antiproliferative effects observed in MCF‐7 at 50 µM, mediated by the negative regulation of genes associated with the cell cycle and metastasis [32]. In addition, Kaempferol has caused a sustained growth arrest of MCF‐7 in dose‐dependent manner at 20, 40, 80 µM [33].

*Apigenin* present in the extracts exhibit specific mechanisms of action that contribute to the overall cytotoxic effect. Recent research has demonstrated its effectiveness against MCF‐7 through the modulation of the PI3K/AKT/p53 pathway at concentrations between 7 and 28 µM [34], as well as its ability to inhibit HeLa cell growth at 37 µM through G1 phase cell cycle arrest [35]. The correlation between the distribution of these bioactive compounds and the observed cytotoxic activity suggests significant synergistic effects, an observation supported by current literature on natural polyphenols. This synergy manifests in a potentiation of the antiproliferative effect when compounds act together, compared to their individual activity, which could explain the notable cytotoxic activity of the studied extracts.

These polyphenols have gained significant relevance in oncological research due to their anticancer properties, favorable bioavailability, ability to overcome drug resistance, and adequate safety profile [36, 37, 38]. This compositional similarity may account for the comparable response observed in the HeLa cell line, suggesting that the parallel behavior could be attributed to the homogeneous distribution of these compounds in the extracts. However, further studies are necessary to confirm this relationship. Based on this analysis, and considering only the qualitative data, we could suggest that the presence of Kaempferol Hexoside‐rhamnoside (**4**) in DA‐Col‐2 may be a key factor in the notable cytotoxic effects observed in MCF‐7 for this extract, which were not evident in DA‐Col‐1. This finding contrasts with the analysis of the DA‐Bra extracts, where only Dihydroxybenzoic acid hexoside (**1**) was common between DA‐Bra‐1 and DA‐Bra‐2. Despite these subtle phytochemical variations, a remarkable difference in cytotoxicity was observed between the extracts. This divergent biological activity could be influenced by the extraction process, as well as by non‐quantified variations in the concentration or abundance of specific metabolites. In complex natural mixtures, even subtle shifts in the final equilibrium of identical components can significantly modulate their interaction with cancer cell lines [39], leading to the distinct biological potencies observed here.

Furthermore, Unidentified minor peaks suggest that geographic origin introduces subtle micro‐compositional variations. In natural products, such trace constituents can significantly influence biological activity [39], either through intrinsic cytotoxicity or by modulating cell susceptibility to major compounds. Therefore, these qualitative differences likely contribute to the divergent cytotoxicity and selectivity profiles observed between DA‐Col and DA‐Bra, highlighting the importance of future guided isolation studies to characterize these specific active molecules.

Although the absence of significant activity against the SiHa cell line could be considered a limitation, this result does not diminish the therapeutic potential of the extracts but rather suggests specificity in their mechanism of action. The IC_50_ values, which were below 5 µg/mL reinforces the potency of these extracts, especially considering that these concentrations are significantly lower than those necessary to affect control cells. The differences observed in cytotoxic activity and selectivity among the evaluated extracts suggest that the composition and relative concentration of bioactive compounds present could be related to their differential effect on the studied cancer cell lines.

However, subsequent research has shown that these cell lines, despite their distinct tissue origin, can present similar responses to specific cytotoxic agents, although with variations in their sensitivity [40, 41]. This apparent contradiction suggests the existence of both shared and specific response mechanisms for each cell line, which is consistent with the differential results observed in our study. For example, the cytotoxicity of biflavonoids isolated from *Araucaria hunsteinii* provides relevant additional evidence, demonstrating that the inhibitory activity depends on the specific position of hydroxyl groups in biflavonoid structures [42]. This finding provides a mechanistic basis for interpreting our results, suggesting that the phytochemical distribution observed in the extracts and the specific arrangement of different chemical substituents such as hydroxyl groups in the identified flavonoids contribute to the observed selective cytotoxic effects.

In this context, the specific biochemical responses of the studied cell lines correlate with previous research. It has been reported that distinctive patterns in NF‐κB pathway modulation occur in HeLa and MCF‐7 cells exposed to hydrogen peroxide (H_2_O_2_) and tumor necrosis factor alpha (TNF‐α). Their findings revealed that MCF‐7 cells exhibit greater c‐Rel activation, while in HeLa cells this activation is regulated by IκB‐ε [43]. These differences evidence distinctive roles of oxidative stress and inflammatory signaling in the progression of each cancer type, suggesting a divergence in their respective biochemical responses.

Although HeLa and SiHa cell lines share similar oncogenic mechanisms, characterized by the expression of viral oncoproteins E6 and E7 of the human papillomavirus (HPV), which inactivate tumor suppressor proteins p53 and pRb, respectively, thus promoting cellular proliferation [44], recent studies reveal significant differences in their molecular behavior. Specifically, these cell lines present notable variations in viral genome integration. The HeLa line contains multiple integrated copies of the HPV‐18 genome, while SiHa possesses approximately one or two copies of HPV‐16 [45]. Such differences in viral load and integration patterns can substantially influence gene expression and treatment responses. Consequently, although a similar response might be expected between both cell lines in extracts DA‐Col‐1 and DA‐Col‐2, the specific molecular factors of each line can generate variations in their behavior. This particularity suggests the need to carefully consider the unique molecular characteristics of each cell line when evaluating the cytotoxic response of the extracts.

Studies on the activity of *D. ambrosioides* extracts in HeLa and MCF‐7 cell lines are limited, however, various studies have demonstrated the effectiveness of its extracts in inhibiting the development and proliferation of other cancer cell lines, including leukemia, hepatocellular carcinoma, and colorectal cancer. In this context, the *in vitro* anticancer potential of kaempferitrin, a flavonol isolated from the ethanolic extract, in human hepatic cancer SMMC‐7721 cell lines, was reported with an IC_50_ of 0.38 µM [13]. This aligns with our findings, where the presence of flavonoids and polyphenols likely drives the observed cytotoxicity. In the same context, potent activity of the aqueous leaf extract against P388 leukemia cells was demonstrated (IC_50_ = 0.105 µg/mL) [14]. Additionally, the methanolic extract significantly suppresses cell proliferation, with IC_50_ values of 129.2, 69.9, and 130.6 µg/mL for Cellosaurus Caco‐2, human colorectal adenocarcinoma HT‐29, and human hepatic cancer HepG2, respectively, where a high concentration phenolic compounds was demonstrated with antioxidant and total phenolic content [46]. In these scarce existing studies, the authors did not report the Selectivity Index (SI), limiting themselves solely to the IC_50_. In our research this parameter is presented, which confers an additional distinctive character, thus confirming its importance and novelty.

In the present study, we observed significant apoptotic activity in the analyzed extracts. The HeLa cell line treated with extract DA‐Col‐1 demonstrated an early apoptosis rate of 31.07%, close to the positive control cisplatin (39.93%), highlighting its potential to induce cellular death. The MCF‐7 cell line exhibited differential sensitivity, with extracts DA‐Col‐2 and DA‐Bra‐2 inducing early apoptosis rates of 34.76% and 31.45%, respectively—notably lower than cisplatin's effect (78.70%). While cisplatin is commonly used for advanced breast cancers with initially favorable responses, resistance development remains a significant challenge [47]. This observation suggests that extracts DA‐Col‐2 and DA‐Bra‐2 may operate through alternative mechanisms, potentially making them valuable candidates for combination therapy approaches to enhance treatment efficacy and overcome resistance.

The observed apoptotic activity also can be linked to specific compounds identified in our extracts. Kaempferol‐3‐O‐rutinoside (**9**), present in extracts DA‐Col‐1, DA‐Col‐2, and DA‐Bra‐2, has been shown to trigger cytoskeleton collapse, mitochondrial dysfunction, and calcium overload, leading to apoptosis in adenocarcinoma [48]. In other hand Isorhamnetin (**17**), identified in extract DA‐Col‐1, exhibits anti‐cancer effects, with ROS‐mediated cell cycle arrest, apoptosis induction, and inhibition of critical signaling pathways such as mTOR, PI3K, MEK1, NF‐κB, and Akt/ERK. It suppresses HIF‐1α expression and arrests the cell cycle at G2/M and S phases while downregulating COX‐2, PI3K, Akt, mTOR, MEK1, and ERKs, and upregulating apoptosis‐related genes (Casp3, Casp9, Apaf1, Bax, and P53) and the mitochondrial apoptosis pathway [49].

Additionally, apigenin 7‐O‐glucoside (**14**), present in extracts DA‐Col‐1 and DA‐Col‐2, has demonstrated the ability to suppress cell proliferation and trigger apoptosis specifically in HeLa cell lines [50]. While the observed apoptotic effects can be associated with these identified compounds, further complementary studies are necessary to confirm the precise mechanisms of action and establish structure‐activity relationships.

## 5 Conclusions

In conclusion, our results demonstrate for the first time the cytotoxic activity of *D. ambrosioides* extracts obtained from two different geographical locations against breast cancer (MCF‐7) and cervical cancer (HeLa) cell lines. These preparations exhibited lower IC_50_ values than those reported in previous studies for other cell lines, indicating enhanced cytotoxic potency. Notably, extracts DA‐Col‐2 and DA‐Bra‐2 displayed high selectivity toward cancer cells while having minimal effects on normal Vero cells, highlighting their potential as less toxic treatment options. Our study emphasized the superior cytotoxic response of the Colombian samples (DA‐Col‐1 and DA‐Col‐2) compared to those obtained from Brazil (DA‐Bra‐1 and DA‐Bra‐2), further confirming the association between geographical location and biological response. We identified important flavonoids such as Kaempferol Hexoside‐rhamnoside (**4**) in DA‐Col‐2, as well as Kaempferol‐3‐O‐rutinoside (**9**), Kaempferol 3‐Lathyroside (**12**), and Quercetin‐3‐O‐α‐L‐rhamnopyranosyl (1‐2)‐β‐D‐glucopyranoside‐7‐O‐α‐L‐rhamnopyranoside (**2**) in DA‐Bra‐2, many of which are well‐known for their anticancer properties and likely responsible for the observed biological effects. The inclusion of information such as the SI and apoptosis induction reinforces the relevance and novelty of this work. Future research directions should include the deep investigation of the specific intracellular signaling pathways, evaluation of efficacy *in vivo* models, and the optimization of extraction methods to improve standardization and activity of the extracts. Consequently, future research should focus on isolating the specific minor constituents that could drive these geographically‐dependent cytotoxic profiles. Overall, *D. ambrosioides* represents a promising source of compounds with therapeutic potential for oncological applications.

## Author Contributions

**A.C.M.A., L.M.T.C., J.W, E.O., D.P, M.A., and S.C.B.G**.: data collection, design, and formal analysis. **A.C.M.A. and L.M.T.C**.: wrote the article. **S.C.B.G., D.P., and E.O**.: revised the article. All authors have reviewed and approved the final article version for submission.

## Conflicts of Interest

The authors declare no conflicts of interest.

## Supporting information




**Supplementary File 1**: cbdv71452‐sup‐0001‐SuppMat.pdf

## Data Availability

Research data are not shared.
