# Supplementary material for: Comparative Evaluation of the Cytotoxic Effect of Dysphania ambrosioides Extracts Against Human Breast and Cervical Cancer Cells
Source: Chem Biodivers. 2026 Jul 9;23(7):e71452. doi: 10.1002/cbdv.71452 (PMC13348732; doi:10.1002/cbdv.71452)
Supplement: Supplementary file 1 — cbdv71452‐sup‐0001‐SuppMat.pdf [file CBDV-23-e71452-s001.pdf]

Supplementary material

Abstract

Dysphania ambrosioides, a prominent medicinal plant, exhibits diverse biological properties with emerging potential in cancer research. Despite its therapeutic promise, the relationship between phytochemical composition and biological activities remains insufficiently explored, particularly regarding geographical variation. This study investigates the correlation between the phytochemical profiles of *D. ambrosioides* extracts from Colombia (DA-Col) and Brazil (DA-Bra) and their cytotoxic activity against HeLa, SiHa, and MCF-7 cancer cell lines.

Qualitative chemical profiling revealed distinct presence/absence patterns of specific secondary metabolites between the two geographical sources. These geographic-driven variations in the phytochemical profile directly influenced the observed biological activity where the extracts exhibited differential cytotoxicity patterns. Specifically, Colombian extract DA-Col-1 showed significant activity against HeLa cells (IC_{50} = 3.09 µg/mL ± 0.32) and DA-Col-2 demonstrated potent effects on MCF-7 cells (IC_{50} = 2.08 µg/mL ± 0.58). Brazilian extract DA-Bra-2 selectively inhibited MCF-7 proliferation (IC_{50} = 3.09 µg/mL ± 0.12). Apoptosis assays indicated that these extracts might induce early apoptotic events, suggesting a potential mechanism of action that warrants further investigation. This study establishes a direct link between the chemical composition of geographically distinct *D. ambrosioides* samples and their selective anticancer potential, providing a foundation for natural product-based drug development targeting specific cancer types.

Keywords: *Natural products, Cytotoxicity, Apoptosis, Phytochemistry.*

Experimental conditions

Extracts analysis was carried out using a UHPLC-QTOF-MS system, UHPLC (Shimadzu-Nexera x2,) equipped with a Shim-pack XR-ODS III column (2.0 × 50 mm, 1.6 µm) from Shimadzu thermostated at 35 °C coupled to the QTOF-MS mass analyzer (Impact II, Bruker Daltonics). The QTOF-MS system was equipped with an electrospray ionization (ESI) source, operating in positive and negative ionization mode. The adopted elution gradient mode, the mobile phase consisted of A: methanol (0.1% formic acid) and B: aqueous phase (0.1% formic acid), The elution gradient started at 5% of A maintained for 2 min, increased to 95% in the next 10 min, and kept for 3 min. Then 95% A linearly decreased to 5% in 2 min, kept for 5 min. The flow rate was 0.3 mL min−1 and the injection volume was 5 µL. The operation parameters of ESI were the following: negative mode, capillary voltage, 2500 V; end plate offset, 500 V; nebulizer pressure, 3 bar (N2); drying gas, 9 L min−1 (N2); and drying temperature, 200 °C. In positive mode, capillary voltage, 4000 V; end plate offset, 500 V; nebulizer pressure, 3 bar (N2); drying gas, 9 L min−1 (N2); and drying temperature,

190 °C. The QTOF-MS system was operating in broadband collision-induced dissociation (bbCID) acquisition mode and recorded spectra over the range m/z 55−1600 with a scan rate of 2 Hz. This mode provides MS and MS/MS spectra at the same time, working at two different collision energies; at low collision energy (10 eV), MS spectra were acquired. At high collision energy (70 eV), no isolation is taking place at the quadrupole, and the ions from the preselected mass range are fragmented at the collision cell. A QTOF-MS external calibration was performed before each injection with a sodium formate solution. Data treatment was processed with Data Analysis 4.2 Software. Besides the accurate mass measurement (error < 5 ppm).

HPLC analysis Chromatograms

Electrospray Ionization Positive Mode (ESI+)

Figure S1. Overlaid HPLC chromatograms of the extracts in ESI+ mode.

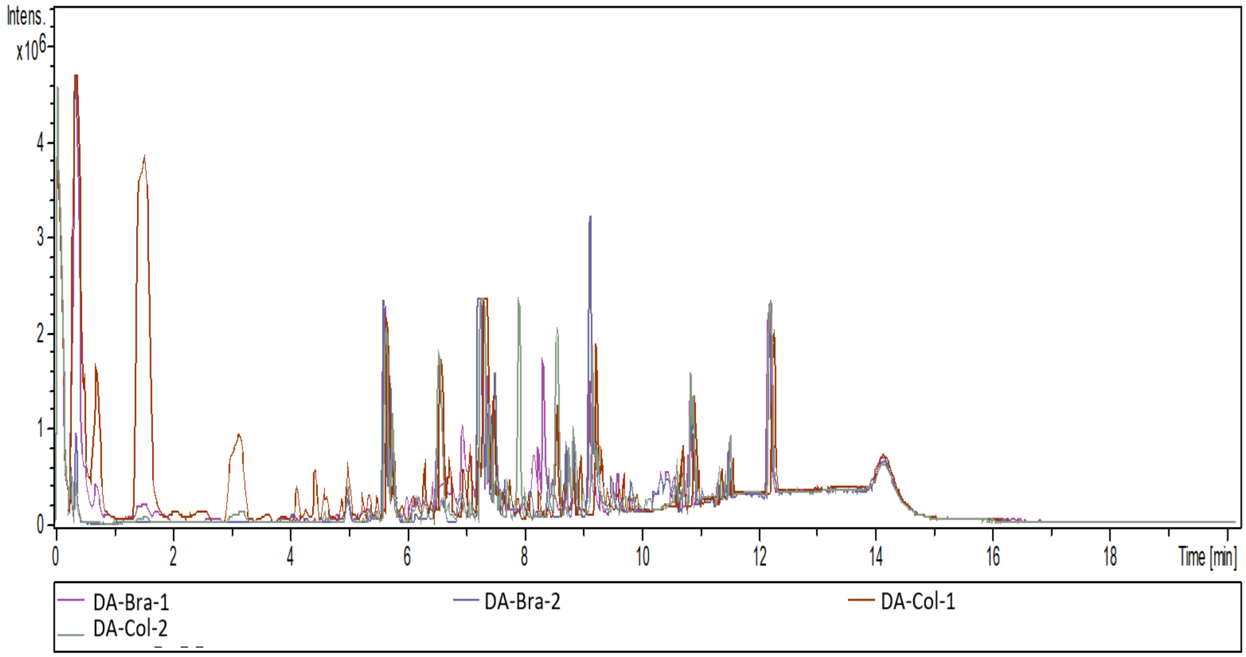

Comparative profile grouping the signals of all analyzed samples under identical chromatographic conditions.

Figure S2. Individual HPLC chromatograms of each extract in ESI+ mode. **A)** DA-Col-1 **B)** DA-Col-2 **C)** DA-Bra-1 **D)** Da-Bra-2)

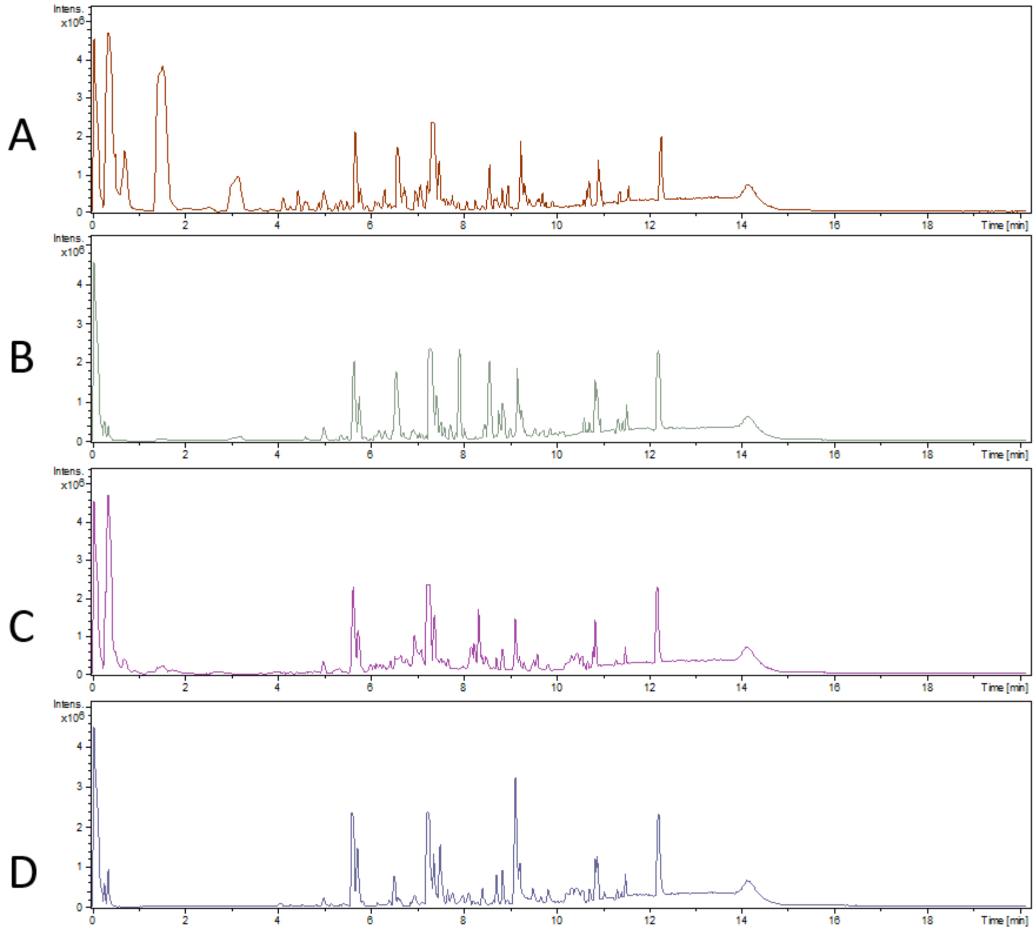

Individual HPLC chromatograms of each extract in ESI− mode.

Electrospray Ionization Negative Mode (ESI−)

The following chromatographic profiles were obtained in ESI− mode, providing complementary data for analyte identification.

Figure S3. Overlaid HPLC chromatograms of the extracts in ESI− mode.

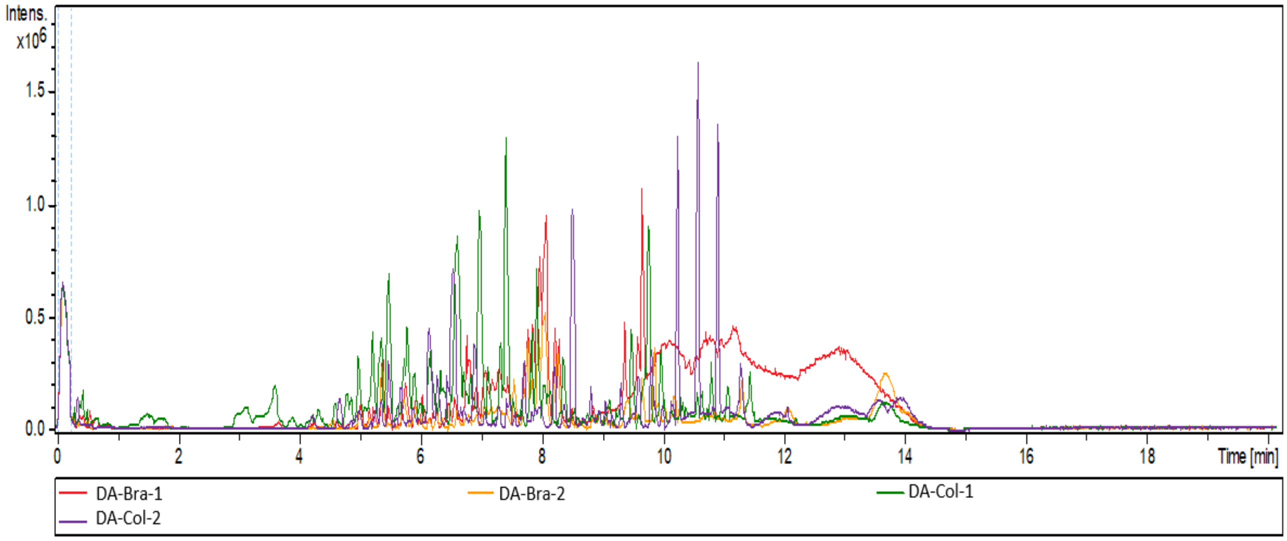

Comparative profile grouping the signals of all analyzed samples under identical chromatographic conditions.

Figure S4. Chromatogram of each extract individually in ESI - mode. A) DA-Col-1 B) DA-Col-2 C) DA-Bra-1 D) Da-Bra-2).

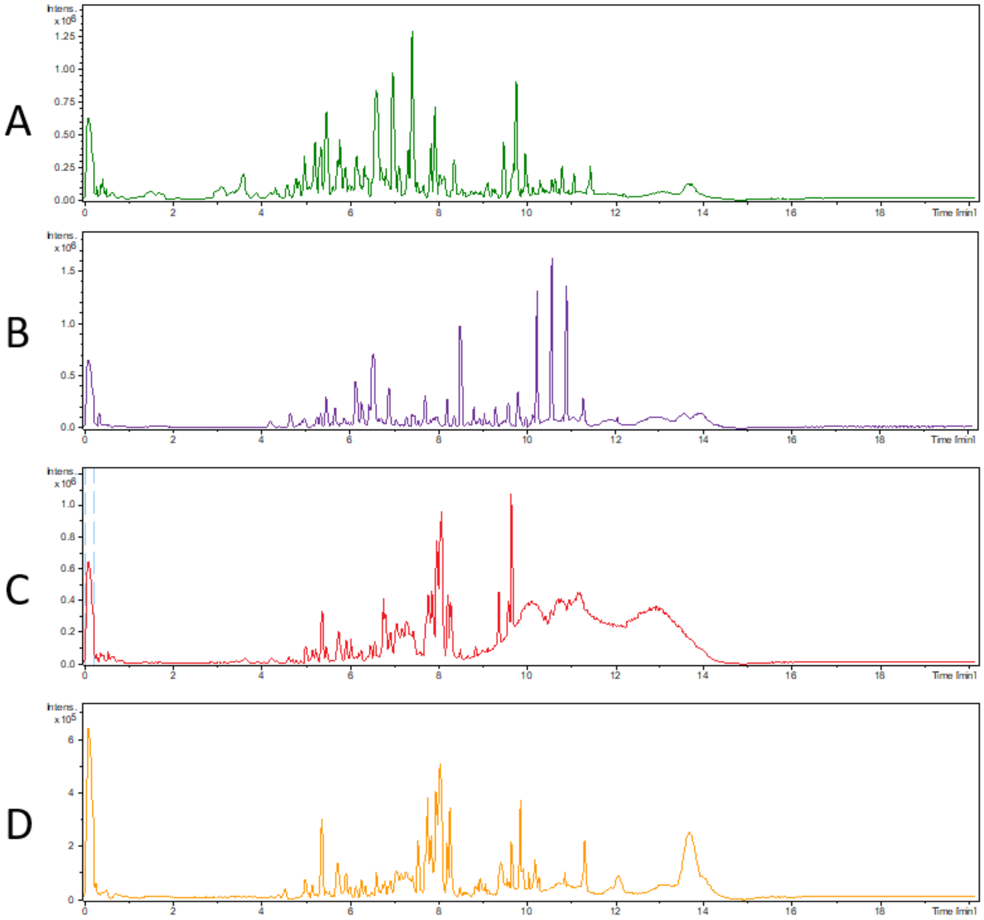

Individual HPLC chromatograms of each extract in ESI− mode.
